# Persistence of chloroquine-resistant haplotypes of *Plasmodium falciparum* in children with uncomplicated Malaria in Lagos, Nigeria, four years after change of chloroquine as first-line antimalarial medicine

**DOI:** 10.1186/s13000-015-0276-2

**Published:** 2015-04-28

**Authors:** Oladosu O Oladipo, Oyibo A Wellington, Colin J Sutherland

**Affiliations:** ANDI Centre of Excellence for Malaria Diagnosis/WHO-FIND Malaria Specimen Collection Site, College of Medicine, University of Lagos, P.M.B 12003, Idiaraba, Lagos, Nigeria; Faculty of Infectious and Tropical Diseases, London School of Hygiene and Tropical Medicine, London, UK

**Keywords:** Chloroquine, *Pfcrt*, *Pfmdr1*, Mutations, Haplotypes, Chloroquine-resistant *Plasmodium falciparum*, Artemesinin combination therapies (ACTs)

## Abstract

**Background:**

In Nigeria, despite the change in National malaria drug policy to artemisinin combination therapy (ACT) in 2005 due to widespread chloroquine resistance, chloroquine (CQ) is still widely used in the treatment of malaria because it is cheap, affordable and accessible. The use of ACT for the management of uncomplicated malaria is currently being promoted. The employment of genetic markers to track circulating chloroquine-resistant parasites are useful in elucidating likely poor efficacy of chloroquine, especially in settings where it is not recommended for the treatment of uncomplicated falciparum malaria. This study determined the prevalence of *pfcrt* haplotypes and point mutations in *pfmdr1* genes four years after the change in antimalarial treatment policy from CQ to the ACTs in Lagos, a commercial city in South-West, Nigeria.

**Methods:**

This was a cross sectional study on uncomplicated malaria in children less than 12 years that presented with fever and other symptoms suggestive of malaria. Parasite DNA was extracted from 119 patients out of 251 children who were positive for *Plasmodium falciparum* by microscopy and amplified. The occurrence of haplotypes was investigated in *pfcrt* gene using probe-based qPCR and single nucleotide polymorphisms in *pfmdr1* gene using nested PCR.

**Results:**

One hundred and nine (109) of the 119 children with P falciparum infection (91.6%) harbourd parasites with the mutant pfcrt haplotype (CVIET). Out of this, 4.2% comprised a mixture of genotypes encoding CVMNK and CVIET, while 4.2% had the wild type (CVMNK). Furthermore, the frequency of point mutations in *pfmdr1* was 62.2% and 69.0% for codons Y86 and F184 respectively. There were no mutations at codons 1034, 1042 and 1246 of the *Pfmdr1* genes.

**Conclusion:**

The high frequency of the CQ-resistant haplotypes (CVIET) and mutations in Pfmdr1 associated with CQ resistance in P. falciparum among these children suggest that CQ-resistant parasites are still in circulation. Continuous use of chloroquine may continue to increase the level of mutations in pfcrt and pfmdr1genes. There is need to strengthen current case management efforts at promoting ACT use as well as urgently restricting access to chloroquine by the National drug regulatory agency, National Agency for Food Drug Administration and Control (NAFDAC).

**Virtual Slides:**

The virtual slide(s) for this article can be found here: http://www.diagnosticpathology.diagnomx.eu/vs/2069472010142303

## Background

The malaria parasite *Plasmodium falciparum* is one of the major causes of morbidity and mortality in sub-Saharan African countries, especially in children and pregnant women. Many factors have contributed to the development and spread of drug resistance, including gene mutations and drug pressure [1]. Resistance to chloroquine (CQ), the most widely used and affordable antimalarial drug, has contributed to the increased in mortality and morbidity caused by *P. falciparum* infections in endemic areas [2]. Resistance to chloroquine, the cheapest and most widely available anti-malarial, has reached significantly high levels leading to replacement with artemisinin-based combination therapy (ACT) in many malaria-endemic countries [3].

Molecular genotyping and characterization of mutations for single nucleotide polymorphism (SNPs) have been used for drug resistance monitoring and could predict emerging or existing drug resistance patterns. Genetically, chloroquine and amodiaquine resistance has been linked to *P. falciparum* chloroquine resistance transporter (*Pfcrt*) gene from different parts of the world [4-7], as well as mutations in *P. falciparum* multidrug resistance 1 (*Pfmdr1*) [8,9].

Polymorphisms in the *Pfmdr*1 gene have been shown by transfection to modulate higher levels of chloroquine resistance and to affect mefloquine, halofantrine, and quinine resistance [10-12]. The role played by *Pfmdr1* mutations (N86Y, Y184F, S1034C and D1246Y) in mediating *in vivo* and *in vitro* chloroquine resistance has received a lot of research interest [13-15]. It has been reported that mutations in the region of *Pfcrt* encompassing codons 72 – 76 is a key marker of *P. falciparum* chloroquine resistance [16]. Substitutions in the wild type allele, encoding CVMNK, give rise to several resistant variants, of which the most common are CVIET in South-East Asia and Africa and SVMNT, which has been reported in South America [4] and Asia [17], but rarely in Africa [18]. The change in single codon of *Pfcrt* gene from Lys (K) to Thr (T) at position 76 (K76T) thus is reported to play a decisive role in conferring resistance to chloroquine [4]. Prolonged use of chloroquine monotherapy has imposed high selection pressure, leading to a substantial increase in the prevalence of this marker in parasite populations worldwide.

In Nigeria, despite the change in National malaria drug policy to ACT because of the widespread and high-level clinical failure rate of chloroquine, CQ is still widely used in the treatment of malaria in the country [19], because it is accessible and affordable. Previous studies have reported resistance to chloroquine by a change at position 76 (K76T) in children treated with chloroquine in Lagos, just before the change in policy [20], in Ibadan [21] and in Osogbo [22]. However, in the above studies, CQ was still the first-line antimalarial medicine before a change in policy to ACTs and the children were treated with chloroquine. The studies did not also provide expanded haplotype information on single nucleotide polymorphisms (SNPs). Therefore, this study was carried out to elucidate single nucleotide polymorphisms in *Pfcrt* and the point mutations in *Pfmdr1* genes with the aim of determining the status of CQ-resistant *Plasmodium falciparum* genes in a diverse and highly populated setting in Lagos, Nigeria, in the light of reports on return of CQ sensitive (wild type) parasites in Malawi and Kenya [23,24] after the total removal of CQ from the population.

## Methods

### Study area/sites

This study was conducted at the St. Kizito Primary Health Centre, Lekki, and Massey Street Children’s Hospital Lagos Island, Lagos State, Southwestern Nigeria – a holoendemic area for malaria. Massey children’s clinic attends to outpatients and serves as a referral paediatric centre in Lagos Metropolis. Lagos State is a commercial area with a diverse population of over 20 million people drawn from other states of Nigeria and West Africa. Lagos is located between latitude and longitude; 6°35′N 3°20′E with an altitude of 40 m (131 ft). The average temperature is 26.4°C (80°F) while the range of average monthly temperatures is 29°C to 35°C. Mean relative humidity for an average year is recorded as 84.7% and on a monthly basis it ranges from 80% in March to 88% in June, July, September and October [25].

### Study population

This research was part of a larger cross-sectional study on uncomplicated malaria in febrile children that was conducted between July 2007 and April 2008. Blood samples were collected through finger pricks from 1,211 febrile children (0–12 years) attending the outpatient clinics in both facilities. Thin and thick blood films were prepared and blood drops were spotted on Whatman filter papers. Parasite DNAs were extracted from a cohort of those that were positive by microscopy.

### Malaria microscopy

This was the first step needed to select the dry blood spots in filter paper for the genetic analysis. Consent was obtained from the Parents/Guardian of the children before they were enrolled. Briefly, thick and thin smears were made on the same slide for each child from finger prick using a sterile lancet. Two slides were made for each child. The first slide was the read “(R)” slide (that is the slide that was read), while the other slide was archived “(A)” slide. This is in line with the quality assurance process of the ANDI Centre of Excellence for Malaria Diagnosis, College of Medicine, University of Lagos to ascertain the children who were positive with *Plasmodium falciparum*. Standard malaria microscopy protocol was used in staining the prepared slides. Parasitaemia levels were obtained from thick smears by counting the number of asexual parasites against 500 leucocytes and expressed per micro liter of blood using an assumed leukocyte count of 8000 wbc/ul. All patients studied received appropriate standard of care after their blood was examined.

### Extraction of DNA from samples collected on filter paper

Parasite genomic DNA was extracted from dried filterpapers using the Chelex®100 method according to methods described elsewhere [26]. Briefly, the discs were lysed in 5% saponin in 1 × PBS and incubated at 37°C overnight. The samples were centrifuged, saponin and debris were removed using a vacuum pump, and the pellets washed twice in buffered saline. The samples were then suspended in 6% Chelex®100 resin and heat-sealed in deep 96-well plates. The samples were incubated in boiling water for 20–25 minutes and then centrifuged to remove resin. Approximately 100 μl of supernatant containing DNA was removed and stored at −20°C.

### Genotyping of the *Pfcrt* locus using qPCR

Double-labelled probes were designed to detecthaplotypes at codons 72 to 76 of the *Pfcrt* gene. Each probe was dual-labelled with a reporter dye at the 5′ end and a quencher moiety at the 3′ end. This method have been previously described using this assay for *Pfcrt* genotyping in the UK [17,27,28]. Briefly, *Pfcrt* DNA was amplified from each sample using previously described conditions and the amplification primers *Pfcrt* F (TGG TAA ATG TGC TCA TGT GTT T) and *Pfcrt* R (AGT TTC GGA TGT TAC AAA ACT ATA GT) [27]. Amplification was performed in a Corbett Rotorgene 3000 (Corbett, Sydney, Australia) in the presence of each of the three double-labelled probes, representing the wild-type and the two most common resistance-associated haplotypes at codons 72–76 of *Pfcrt*. The probes were crt76CVMNK wild-type, 5′FAM-TGT GTA ATG AAT AAA ATT TTT GCT AA-BHQ1 (3D7 DNA from MR4 used as a positive control); crt76CVIET resistant, 5′JOE-TGT GTA ATT GAA ACA ATT TTT GCT AA-BHQ1 (Dd2 DNA from MR4 used as a positive control); and crt76SVMNT resistant, 5′ROX-AGT GTA ATG AAT ACA ATT TTT GCT AA-BHQ2 (7G8 DNA from MR4 used as a positive control). The control parasite DNA was obtained directly from The Malaria Research and Reference Reagent Resource (MR4, Manassas, Vermont, USA). Samples were considered positive for a particular genotype if a CT (threshold cycle) value of 35 cycles or fewer was obtained in at least two independent PCR experiments. Nuclease-free water was included as a negative control.

### *Pfmdr1* genotyping

Amplification of the *Pfmdr1* gene was performed in three fragments (FR1, FR3 and FR4). Primers and cycling conditions used for the three fragments are listed in Table 1. These samples were amplified using Nested PCR reactions. In each reaction, appropriate known positive (Dd2, 7GB and FCR3) and negative samples (DNA negative wells on each row) were used.

**Table 1:** ***Pfmdr1*** **PCR primer sequences and reaction conditions used in Fragments 1, 3 and 4 amplification reactions**

**Gene fragment**	**Primer name**		**Primer sequence**	**Codons**	**PCR cycling conditions**
Fragment 1					
Primary FR1	FN1/1	F	5′- AGGTTGAAAAAGAGTTGAAC-3′	86, 184	94°C 3 min/[94°C 30 s-45°C 60 s 72°C 60 s]
	REV/C1	R	5′- ATGACACCACAAACATAAAT-3′		×30 cycles
Nested FR1	MDR2/1	F	5′- ACAAAAAGAGTACCGCTGAAT -3′		72°C for 5 minutes/15°C 5 min
	NEWREV1	R	5′-AAACGCAAGTAATACATAAAGTC-3′		
Fragment 3					
Primary FR3	MDRFR3N1	F	5′-GCATTTTATAATATGCATACTG-3′	1034, 1042	94°C 3 min/[94°C 30 s-55°C 60 s 65°C 40 s]
	MDRFR3R1	R	5′-GGATTTCATAAAGTCATCAAC-3′		×30 cycles
Nested FR3	MDRFR3N2	F	5′-GGTTTAGAAGATTATTTCTGTA-3′		72°C 5 min/15°C 5 min
	MDRFR3R1	R	5′-GGATTTCATAAAGTCATCAAC-3′		
Fragment 4					
Primary FR4	MDRFR4N1	F	5′- CAAACCAATCTGGATCTGCAGAAG -3′	1246	94°C 3 min/[94°C 30 s-55°C 60 s-65°C 40 s]
	MDRFR4R1	R	5′-CAATGTTGCATCTTCTCTTCC -3′		×30 cycles
Nested FR4	MDRFR4N2	F	5′- GATCTGCAGAAGATTATACTG -3′		72°C 5 min/15°C 5 min
	MDRFR4R1	R	5′- CAATGTTGCATCTTCTCTTCC -3′		

FR - Fragment F - forward R - Reverse.

NB: Cycling conditions are the same for primary and nested PCRs.

The *Pfmdr1* PCR products of nested reactions were separated by gel electrophoresis on a 1.2% agarose gel stained with ethidium bromide to identify amplified bands of DNA under ultra-violet illumination. Amplicons from nested PCR products were purified using ExoSap IT reaction [29] and were sent for sequencing. Sequencing was performed using the BigDye 3.1, Cycle Sequencing Kit (Applied Biosystems, UK) using conditions and sequencing primer pairs described elsewhere [26,30]. The sequence of amplified DNA products was determined using ABI PRISM 3730 Genetic Analyser (Applied Biosystems, UK). Chromas software (Technelysium, Australia) and was used to analyse the sequence results. The DNA sequence was compared with reference sequence of the *Pfmdr1*, portions of the *P. falciparum* 3D7 clone using BLAST similarity alignment (Washington University, USA). In each reaction, appropriate control DNA samples with known *Pfmdr1* sequences were used in parallel with field-collected parasite isolates in every step of the protocol.

## Results

A total of 1,211 children were screened in this study. The children tested included 658 (54.4%) males and 553 (45.6%) females; mean age ± SD was 2.65 ± 2.83; while their mean body temperature was 37.8°C (range, 35.5–42°C). Out of the total children (*<*12 years) tested, 251 (20.7%) were positive for malaria parasites by microscopy. Parasite DNAs were successfully obtained from a cohort of 251 microscopy positive malaria parasites samples during extraction.

### *Pfcrt* polymorphisms

A total of 119 DNA samples were successfully tested for *Pfcrt* genotype at codons 72–76. Most of the isolates (91.6%) harboured parasites with the CVIET haplotypes. The proportion of the wild type (CVMNK) among the isolates was 4.2%, while mixed haplotype infections (CVMNK/CVIET) were found in 5 isolates (4.2%) (Table 2). The Southeast Asian/South American chloroquine-resistant haplotype (SVMNT) was not seen in any of the isolates.

**Table 2:** ***Pfcrt*** **haplotypes and frequency of** ***Pfmdr1*** **codons in Nigerian children**

**Gene**	**n**	**Genotype/haplotype**	**Prevalence (%)**
***Pfcrt***	119	CVIET (mutant type)	109 (91.6)
Amino acids (72–76)		CVMNK (wild type)	5 (4.2)
		CVMNK/CVIET (mixed)	5 (4.2)
***Pfmdr1***			
Amino acids 86, 184, 1034, 1042 and 1246	74	86Y (mutant type)	46 (62.2)
		86 N (wild type)	14 (18.9)
		86 N + 86Y (mixed)	14 (18.9)
	71	184 F (mutant type)	49 (69)
		184Y (wild type)	11 (15.5)
		184 F + 184Y (mixed)	11 (15.5)
	81	1034C (mutant type)	0(0)
		1034S (wild type)	81(100)
	81	1042D (mutant type)	0(0)
		1042 N (wild type)	81(100)
	29	1246S (mutant type)	0(0)
		1246D (wild type)	29(100)

### *Pfmdr1* polymorphisms

The isolates were genotyped for *Pfmdr1* at codons 86, 184, 1034, 1042 and 1246. The majority of isolates had the mutant *Pfmdr1* Y86 and F184 alleles. Of the isolates, 62.2% (46/74) carried the mutant allele Y86 and 69% (49/71) and had the mutant allele F184 (Table 2). There was no mutation in codons 1034, 1042 and 1246 in any isolates.

## Discussion

Self-treatment with chloroquine and other monotherapies are still high in the population because of their affordability, accessibility, and low implementation of the malaria treatment guidelines that recommends the use of ACTs. The access to, and high usage of CQ and other monotherapies in the treatment of malaria makes the determination of chloroquine resistant *Plasmodium falciparum* in Lagos imperative several years after the change in malaria treatment policy. CQ is still sold especially in the informal private sector among the Private Propriety Medicine Vendors (PPMVs), otherwise known as medicine retailers, a group that provide malaria treatment to over 60% of fever patients in the country. Further, the PPMVs sell antimalarial medicines on the basis of their clients’ complaints and are not permitted to do a blood based test due to policy restrictions.

Mutations in the region of *Pfcrt* codons 72 – 76 is said to be a key marker of *P. falciparum* chloroquine resistance [16]. This study showed high prevalence of *Pfcrt* CVIET haplotype (72–76). This result is consistent with previous study where majority (98%) of the isolates genotyped carried the chloroquine resistant CVIET haplotype in Uganda [31] and in Swaziland [32]. Persistence of high prevalence of CVIET was also reported in Ethiopia due the continuous use of chloroquine for the treatment of *P. vivax* [33]. This study showed that the SVMNT haplotype does not occur in Lagos, Nigeria. The absence of the SVMNT haplotype which is known to occur majorly in South America is consistent with many other reports in Africa [16,32] except studies in part of East Africa (Tanzania and Angola)that reported SVMNT [18,34]. This was presumed to be as a result of *P. falciparum* resistance to amodiaquine or its metabolite desethyl-amodiaquine following the use of amodiaquine as monotherapy.

The present study showed prevalence of 62.2% and 69.0% for *Pfmdr1* Y86 and F184 (mutant-type) respectively. The role of *Pfmdr1* gene mutations in anti-malarial drugs resistance is still controversial. An *in vivo* study where a chloroquine-resistant infection was reported showed absence of mutations at codons *Pfmdr1* 86 and 1246 in the [35,36]. Similarly, a study in South-Eastern Iran reported a strong association between *pfcrt* K76T, but not *pfmdr1* N86Y mutation and *in vivo* chloroquine resistance [37]. A study in Haiti reported mutation in F184 only in the *Pfmdr1* gene and no mutation was seen in *Pfcrt* gene codon 72–76 [38]. In contrast, Y86 have been reported to be responsible for chloroquine resistance in combination with *Pfcrt* 76 T [13,21,39] and another study from Madagascar reported an association between *Pfmdr1* Y86 mutant alleles and chloroquine clinical resistance with no such association with *Pfcrt*gene [40]. Polymorphisms in the *Pfmdr1* gene have been said to be under artemether-lumenfantrine selection pressure [41]. Selection of *Pfmdr1* Y86by amodiaquine and chloroquine were reported previously in the Gambia [42], and in Kenya [43]. Thus, the success of treatment with ACTs may largely depend on the parasite’s existing level of tolerance to the partner drugs.

Some earlier studies in the pre-ACT days in South-West Nigeria reported high chloroquine-resistant parasites in children treated with CQ when CQ was the drug of choice for the treatment of malaria and they only determined mutation at position 76 (K76T). Mutation at 76 (K76T) had been reported to play a decisive role in conferring resistance to CQ [4]. In a semi-urban area of Lagos, South-West Nigeria, the prevalence of T76 mutation was 74.6% [20], while another study In Ibadan; South-West Nigeria reported 62% and 29% for T76 and Y86 mutations respectively [21]. Prevalence of 74%, 29% and 64% were reported for mutations at T76, Y86 and F184 respectively in children with *P. falciparum* even before they were treated in Osogbo, South-West Nigeria while in another study in Ibadan (south-West Nigeria), a prevalence of 60%, 33% and 14% mutations at T76, Y86 and F184 respectively was reported in children whose age ranged from 6 month - 12 years [44]. Also another study in Ogun State, still in the South-West zone of Nigeria, reported a prevalence of 96.9% at K76T among children under the age of five years [45].

Our study in Lagos, South-West Nigeria, has showed the persistence of chloroquine-resistant parasites circulating in children four years after the change in policy for the treatment of uncomplicated malaria from CQ to ACTs. It is therefore important that apart from the change in policy to ACTs, there is an urgent need to restrict the use of chloroquine in the general population by the regulatory agency for drugs. Since resistant phenotypes often have fitness costs [46], their prevalence is likely to decline after removal of the selective pressure. In countries where the change in policy from chloroquine to ACT was strictly enforced, marked decrease in chloroquine-resistant parasites in the population was recorded. In a recent surveillance study in Honduras, Central America, where CQ is still used for the management of uncomplicated malaria, all the samples tested showed CQ susceptibility in the *Pfcrt* “CVMNK” genotype in codons 72–76 [47].

There was a decrease in the frequency of *Pfcrt*76T mutation when CQ was abolished in the treatment of *P. falciparum* malaria in the People’s Republic of China [48]; prevalence of mutant alleles of *Pfcrt*76T decreased from 64.5% in 2002 to 16% in 2004 and that of the mutant *Pfmdr1* 86Y alleles decreased from 46.6% to 2.7% two and half years after successful withdrawal of CQ in coastal Tanzania [49]. It was also reported that the prevalence of the CQ-resistant *Pfcrt*76T genotype decreased from 85% in 1992 to 13% in 2000 in Malawi [49]. In 2001, CQ cleared 100% of 63 asymptomatic *P. falciparum* infections as no isolates were resistant to CQ *in vitro*, and no infections with the CQ resistant *Pfcrt*76T genotype were detected [50]. Similarly, it was shown that CQ was again an efficacious treatment for malaria, 12 years after it was successfully withdrawn from use in Malawi [23]. Similar result was also reported in Kenya where the frequency of the *Pfcrt*-76 mutant significantly decreased from around 95% to 60%, though, the frequency of *Pfmdr1*-86 did not decline substantially [24]. In Tanzania, where chloroquine is no longer in use, the frequency of the wild type CVMNK haplotype increased from 6% in 2003 to 30% in 2007. These findings may reflect decreasing drug pressure of chloroquine on the parasite populations in these areas.

Surveillance for antimalarial drug resistance, using the platform provided by the National Malaria control Programme in Nigeria should be supported to undertake regular and robust monitoring of malaria parasite resistance genes for trends. The use of amodiaquine has been associated to a certain extent with *Pfcrt*76T and *Pfmdr1* 86Y mutations [21,43]. Furthermore, the partner drugs to ACT are also threatened by the development of resistance if treatment of malaria with antimalarial monotherapy is not abolished [51,52]. Importantly, the implementation of the current malaria treatment policy using the ACTs should be strengthened, vigorously promoted, through regular training, supervision among health workers; and the institution of best procurement practices for malaria medicines at all levels based on National Policy recommendations. The regulatory agency, National Agency for Food Drug Administration and Control (NAFDAC) should regulate access to chloroquine in Nigeria while the general public should be enlightened on the high levels of circulating resistant-malaria parasite genes to chloroquine, its low efficacy and to discourage its continuous use for the for the treatment of uncomplicated malaria. Access to ACTs should be expanded and made affordable especially in the private sector where a high number of persons with fever are treated.

## Conclusion

There is a high level of CQ-resistant-haplotypes of *P. falciparum* (CVIET) and high frequency of mutations in *Pfmdr1* four years after the change in malaria treatment policy from CQ to ACTs for the treatment of uncomplicated malaria in Lagos, South-western Nigeria. This suggests persistent circulation and spread of CQ-resistant *P. falciparum* parasites in the population and the need to strengthen current efforts at promoting ACT use in the treatment of uncomplicated malaria. The continued use of CQ for the treatment of malaria in Nigeria could be one major reason for the persistence of mutant *Pfcrt* haplotypes and *Pfmdr1* mutations in the study area. This could threaten the efficacy of partner drugs in the ACTs. Data from this study provided evidence of continued CQ use and the need for the key players in the Federal Ministry of Health to decisively regulate the use of CQ in Nigeria.

### Ethical approval

This study was approved by The Research, Grants and Experimentation Committee, of the College of Medicine, University of Lagos, Idi-Araba, Lagos, Nigeria and the Research Ethics Committee of the Lagos University Teaching Hospital, Idi-Araba Lagos, Nigeria.

## Abbreviations

CQ: Chloroquine
ACT: Artemisinin combination therapy
*Pfcrt*: *Plasmodium falciparum* chloroquine resistant transporter
*Pfmdr1*: *Plasmodium falciparum* multidrug resistant
PPMVs: Private Propriety Medicine Vendors
qPCR: Quantitative polymerase chain reaction
DNA: Deoxyribonucleic acid
CT: Threshold cycle

## References

[1] Talisuna AO, Nalunkuma-Kazibwe A, Langi P, Mutabingwa TK, Watkins WW, Van Marck E (2004). Two mutations in dihydrofolatereductase combined with one in the dihydropteroate synthase gene predict sulphadoxine-pyrimethamine parasitological failure in Ugandan children with uncomplicated falciparum malaria. Infect Genet Evol.

[2] WHO (2010). Guidelines for the treatment of malaria.

[3] WHO (2001). Antimalarial drug combination therapy: report of a WHO technical consultation.

[4] Fidock DA, Nomura T, Talley AK, Cooper RA, Dzekunov SM, Ferdig MT (2000). Mutations in the *P. falciparum* digestive vacuole transmembrane protein PfCRT and evidence for their role in chloroquine resistance. Mol Cell.

[5] Chen N, Kyle DE, Pasay C, Fowler EV, Baker J, Peters JM (2003). *Pfcrt* allelic types with two novel amino acid mutations in chloroquine-resistant *Plasmodium falciparum* isolates from the Philippines. Antimicrob Agents Chemother.

[6] Djimde AA, Doumbo OK, Traore O, Guindo AB, Kayentao K, Diourte Y (2003). Clearance of drug-resistant parasites as a model for protective immunity in Plasmodium falciparum malaria. Am J Trop Med Hyg.

[7] Ursing J, Kofoed PE, Rodrigues A, Rombo L, Gil JP (2007). *Plasmodium falciparum* genotypes associated with chloroquine and amodiaquine resistance in Guinea-Bissau. Am J Trop Med Hyg.

[8] Foote SJ, Thompson JK, Cowman AF, Kemp DJ (1999). Amplification of the multidrug resistance gene in some chloroquine-resistant isolates of P. falciparum. Cell.

[9] Hayward R, Saliba KJ, Kirk K (2005). Mutations in pfmdr1 modulate the sensitivity of Plasmodium falciparum to the intrinsic antiplasmodial activity of verapamil. Antimicrob Agents Chemother.

[10] Djimde A, Doumbo OK, Cortese JF, Kayentao K, Doumbo S, Diourte Y (2001). A molecular marker for chloroquine-resistant falciparum malaria. N Engl J Med.

[11] Sidhu AB, Verdier-Pinard D, Fidock DA (2002). Chloroquine resistance in *Plasmodium falciparum* malaria parasites conferred by *Pfcrt*mutations. Science.

[12] Cheruiyot J, Ingasia LA, Omondi AA, Juma DW, Opot BH, Ndegwa JM (2014). Polymorphisms in *Pfmdr1*, *Pfcrt*, and *Pfnhe1* genes are associated with reduced *In Vitro* Activities of Quinine in *Plasmodium falciparum* Isolates from Western Kenya. Antimic Agents Chemo.

[13] Duraisingh MT, Cowman AF (2005). Contribution of the *pfmdr1*gene to antimalarial drug resistance. Acta Trop.

[14] Khalil IF, Alifrangis M, Tarimo DS, Staalso T, Satti GM, Theander TG (2005). The roles of the pfcrt 76 T and pfmdr1 86Y mutations, immunity and the initial level of parasitaemia, in predicting the outcome of chloroquine treatment in two areas with different transmission intensities. Ann Trop Med Parasitol.

[15] Andriantsoanirina V, Ratsimbasoa A, Bouchier C, Jahevitra M, Rabearimanana S, Radrianjafy R (2009). Plasmodium falciparum drug resistance in Madagascar: facing the spread of unusual pfdhfr and pfmdr-1 haplotypes and the decrease of dihydroartemisinin susceptibility. Antimicrob Agents Chemother.

[16] Gadalla NB, Elzaki SE, Mukhtar E, Warhurst DC, El-Sayed B, Sutherland CJ (2010). Dynamics of pfcrt alleles CVMNK and CVIET in chloroquine-treated Sudanese patients infected with Plasmodium falciparum. Malaria J.

[17] Sutherland CJ, Haustein T, Gadalla N, Armstrong M, Doherty JF, Chiodini PL (2007). Chloroquine resistant *Plasmodium falciparum* infections among UK travellers returning with malaria after chloroquine prophylaxis. J Ant Chem.

[18] Alifrangis M, Dalgaard MB, Lusingu JP, Vestergaard LS, Staalsoe T, Jensen AT (2006). Occurrence of the Southeast Asian/South American SVMNT haplotype of the chloroquine resistance transporter gene in *Plasmodium falciparum* in Tanzania. J Infect Dis.

[19] Ogungbamigbe T, Ogunro P, Elemile P, Egbewale B, Olowu O, Abiodun O (2005). Presciption patterns of antimalarial drugs among medical practitioners in Osogbo Metropolis, South-West Nigeria. Trop Med Health.

[20] Olukosi YA, Iwalokun BA, Magbagbeola EO, Akinwande O, Adewole TA, Agomo PU (2005). Pattern of rural–urban aquisition of Pfcrt T76 allele among Nigerian children with uncomplicated Plasmodium falciparum malaria. Afr J Biotec.

[21] Happi CT, Gbotosho GO, Folarin OA, Bolaji OM, Sowunmi A, Kyle DE (2006). Association between mutations in *plasmodium falciparum* chloroquine resistance transporter and *p. falciparum* multidrug resistance 1 genes and *in vivo* amodiaquine resistance in *p. falciparum* malaria–infected children in Nigeria. Am J Trop Med Hyg.

[22] Ojurongbe O, Ogungbamigbe TO, Fagbenro-Beyioku AF, Fendel R, Kremsner PG, Kun JF (2007). Rapid detection of Pfcrt and Pfmdr1 mutations in Plasmodium falciparum isolates by FRET and in vivo response to chloroquine among children from Osogbo, Nigeria. Malar J.

[23] Laufer MK, Thesing PC, Eddington ND, Masonga R, Dzinjalamala FK, Takala SL (2006). Return of chloroquine antimalarial efficacy in Malawi. N Engl J Med.

[24] Mwai L, Ochong E, Abdirahman A, Kiara SM, Ward S, Kokwaro G (2009). Chloroquine resistance before and after its withdrawal in Kenya. Malaria J.

[25] Nigerian muse Online: 7 February 2007. Available at: http://en.wikipedia.org/wiki/Lagos. Accessed 4 April 2013.

[26] Humphreys GA, Merinopoulos I, Ahmed J, Whitty CJM, Mutabingwa TK, Sutherland CJ (2007). Amodiaquine and artemether-lumefantrine select distinct alleles of the Plasmodium falciparum pfmdr1 gene in Tanzanian children treated for uncomplicated malaria. Antimicrob Agents Chemother.

[27] Sutherland CJ, Fifer H, Pearce RJ, BinReza F, Nicholas M, Haustein T (2009). Novel *pfdhps*haplotypes among imported cases of *Plasmodium falciparum* malaria in the UK. Antimicrob Agents Chemother.

[28] Wilson PE, Kazadi W, Kamwendo DD, Mwapasa V, Purfield A, Meshinick SR (2005). Prevalence of pfcrt mutations in Congolese and Malawian Plasmodium falciparum isolates as determined by a new Taqman assay. Acta Trop.

[29] Bell J (2008). A simple way to treat PCR products prior to sequencing using ExoSAP-IT. Biotechniques.

[30] Pearce RJ, Drakeley C, Chandramohan D, Mosha F, Roper C (2003). Molecular determination of point mutation haplotypes in the dihydrofolatereductase and dihydropteroate synthase of *Plasmodium falciparum* in three districts of northern Tanzania. Antimicrob Agents Chemother.

[31] Keen J, Farcas GA, Zhong K, Yohanna S, Dunne MW, Kain KC (2007). Real-time PCR assay for rapid detection and analysis of PfCRT haplotypes of chloroquine resistant *Plasmodium falciparum* isolates from India. J Clin Microbiol.

[32] Dlamini SV, Beshir K, Sutherland CJ (2010). Markers of anti-malarial drug resistance in *Plasmodium falciparum* isolates from Swaziland: identification of *pfmdr1*-86 F in natural parasite isolates. Malaria J.

[33] Golassa L, Enweji N, Erko B, Aseffa A, Swedberg G (2014). High prevalence of pfcrt-CVIET haplotype in isolates from asymptomatic and symptomatic patients in south-central Oromia, Ethiopia. Malar J.

[34] Gama BE, Pereira de Carvalho GA, LutucutaKosi FJ, Almeida de Oliveira NK, Fortes F, Rosenthal PJ (2010). Plasmodium falciparum isolates from Angola show the StctVMNT haplotype in the pfcrt gene. Malar J.

[35] Basco LK, Bras JL, Rhoades Z, Wilson CM (1995). Analysis of pfmdr1 and drug susceptibility in fresh isolates of *Plasmodium falciparum* from Sub-Saharan Africa. Mol Biochem Parasitol.

[36] von Seidlein L, Duraisingh MT, Drakeley CJ, Bailey R, Greenwood BM, Pinder M (1997). Polymorphism of the pfmdr1 gene and chloroquine resistance in *Plasmodium falciparum* in the Gambia. Trans R Soc Trop Med Hyg.

[37] Zakeri S, Afsharpad M, Kazemzadeh T, Mehdizadeh K, Shabani A, Djadid ND (2008). Association of pfcrt but not pfmdr1 alleles with chloroquine resistance in Iranian isolates of *Plasmodium falciparum*. Am J Trop Med Hyg.

[38] ElBadry ME, Existe A, Victor YS, Memnon G, Fukuda M, Dame JB (2013). Survey of Plasmodium falciparum multidrug resistance-1 and chloroquine resistance transporter alleles in Haiti. Malar J.

[39] Nagesha HS, Din S, Casey GJ, Susanti AI, Fryauff DJ, Reeder JC (2001). Mutations in the *pfmdr1, dhfr*and *dhps* genes of *Plasmodium falciparum* are associated with *in-vivo* drug resistance in west Papua, Indonesia. Trans R Soc Trop Med Hyg.

[40] Andriantsoanirina V, Ratsimbasoa A, Bouchier C, Tichit M, Jahevitra M, Rabearimanana S (2010). Chloroquine clinical failures in *Plasmodium falciparum* malaria are associated with mutant pfmdr-1, not pfcrt in Madagascar. PLoS One.

[41] Happi CT, Gbotosho GO, Folarin OA, Sowunmi A, Hudson T, O’Neil M (2009). Selection of Plasmodium falciparum multidrug resistance gene 1 alleles in asexual stages and gametocytes by artemether-lumefantrine in Nigerian children with uncomplicated falciparum malaria. Antimic Agents Chemoth.

[42] Duraisingh MI, Drakeley CI, Muller O, Bailey R, Snounou G, Targett GA (1997). Evidence for selection for the tyrosine 86 allele of *pfmdr1* gene of *Plasmodium falciparum* by chloroquine and amodiaquine. Parasitology.

[43] Holmgren G, Gil GP, Ferreira PM, Veiga MI, Obonyo CO, Bjorkman A (2006). Amodiaquine resistant *Plasmodium falciparum* malaria invivo is associated with selection of *Pfcrt*76T and *Pfmdr*1 86Y. Infect Genet Evol.

[44] Folarin OA, Gbotosho GO, Sowunmi A, Olorunsogo OO, Oduola AMJ, Happi TC (2008). Chloroquine resistant *Plasmodium falciparum* in Nigeria: relationship between *pfcrt*and *pfmdr1* polymorphisms, *In-Vitro* resistance and treatment outcome. Open Trop Med J.

[45] Efunshile M, Runsewe-Abiodun T, Ghebremedhin B, Konig W, Konig B (2011). Prevalence of the molecular marker of chloroquine resistance (pfcrt 76) in Nigeria 5 years after withdrawal of the drug as first-line antimalarial. A cross-sectional study. SAJCH.

[46] Yang Z, Zhang Z, Sun X, Wan W, Cui L, Zhang X (2007). Molecular analysis of chloroquine resistance in *Plasmodium falciparum*in Yunnan Province, China. Trop Med Intl Health.

[47] Fontecha GA, Sanchez AL, Mendoza M, Banegas E, Mejía-Torres RE (2014). A four-year surveillance program for detection of *Plasmodium falciparum* chloroquine resistance in Honduras. MemInst Oswaldo Cruz.

[48] Wang X, Um J, Li Q, Chen P, Guo X, Fu L (2005). Decreased prevalence of the *Plasmodium falciparum* chloroquine resistance transporter 76 T marker associated with cessation of choroquine use against *P. falciparum* malaria in Hainan, people’s Republic of China. Am J Trop Med Hyg.

[49] Temu EA, Kimani I, Tuno N, Kawada H, Minjas JN, Takagi M (2006). Monitoring chloroquine resistance using *Plasmodium falciparum* parasites isolated from wild mosquitoes in Tanzania. AmJ Trop Med Hyg.

[50] Kublin JG, Cortese JF, Njunju EM, Mukadam RA, Wirima JJ, Kazembe PN (2003). Reemergence of chloroquine-sensitive *Plasmodium falciparum* malaria after cessation of chloroquine use in Malawi. J Infect Dis.

[51] Noedl H, Se Y, Schaecher K, Smith BL, Socheat D, Fukuda MM (2008). Artemisinin Resistance in Cambodia 1 (ARC1) Study Consortium: evidence of artemisinin-resistant malaria in western Cambodia. N Eng J Med.

[52] Dondorp AM, Nosten F, Yi P, Das D, Phyo AP, Tarning J (2009). Artemisinin resistance in Plasmodium falciparum malaria. N Eng J Med.

